# 3D Printing/Additive Manufacturing Single Titanium Dental Implants: A Prospective Multicenter Study with 3 Years of Follow-Up

**DOI:** 10.1155/2016/8590971

**Published:** 2016-05-29

**Authors:** Samy Tunchel, Alberto Blay, Roni Kolerman, Eitan Mijiritsky, Jamil Awad Shibli

**Affiliations:** ^1^Private Practice, Rua Asia 173, Cerqueira Cesar, 05413-030 Sao Paulo, SP, Brazil; ^2^Private Practice, Rua Inacio Pereira da Rocha 147, 05432-010 Sao Paulo, SP, Brazil; ^3^Department of Periodontology, The Maurice and Gabriela Goldschleger School of Dental Medicine, University of Tel Aviv, Ramat-Aviv, 6997801 Tel Aviv, Israel; ^4^Department of Oral Rehabilitation, The Maurice and Gabriela Goldschleger School of Dental Medicine, University of Tel Aviv, Ramat-Aviv, 6997801 Tel Aviv, Israel; ^5^Department of Periodontology and Oral Implantology, Dental Research Division, Guarulhos University, Praca Teresa Cristina 229, 07023070 Guarulhos, SP, Brazil

## Abstract

This prospective 3-year follow-up clinical study evaluated the survival and success rates of 3DP/AM titanium dental implants to support single implant-supported restorations. After 3 years of loading, clinical, radiographic, and prosthetic parameters were assessed; the implant survival and the implant-crown success were evaluated. Eighty-two patients (44 males, 38 females; age range 26–67 years) were enrolled in the present study. A total of 110 3DP/AM titanium dental implants (65 maxilla, 45 mandible) were installed: 75 in healed alveolar ridges and 35 in postextraction sockets. The prosthetic restorations included 110 single crowns (SCs). After 3 years of loading, six implants failed, for an overall implant survival rate of 94.5%; among the 104 surviving implant-supported restorations, 6 showed complications and were therefore considered unsuccessful, for an implant-crown success of 94.3%. The mean distance between the implant shoulder and the first visible bone-implant contact was 0.75 mm (±0.32) and 0.89 (±0.45) after 1 and 3 years of loading, respectively. 3DP/AM titanium dental implants seem to represent a successful clinical option for the rehabilitation of single-tooth gaps in both jaws, at least until 3-year period. Further, long-term clinical studies are needed to confirm the present results.

## 1. Introduction

Dental implants available for clinical uses are conventionally produced from rods of commercially pure titanium (cpTi) or its alloy Ti-6Al-4V (90% titanium, 6% aluminium, and 4% vanadium). Manufacturing processes involve machining, at a later stage, postprocessing with application of surface treatments, with the aim of enhancing healing processes, and osseointegration around dental implants [1, 2].

Over the last years, several surface treatments have been proposed, such as sandblasting, grit-blasting, acid-etching, and anodization; deposition of hydroxyapatite, calcium-phosphate crystals, or coatings with other biological molecules are all examples of attempts to obtain better implant surfaces [2–4]. In fact, several* in vitro* studies have identified that rough implant surfaces can positively influence cell behaviour and therefore bone apposition, when compared to smooth surfaces [3, 5]. Rough surfaces show superior molecules adsorption from biological fluids, improving early cellular responses, including extracellular matrix deposition, cytoskeletal organization, and tissues maturation. This implant surface topography can finally lead to a better and faster bone response around rough surfaced dental implants [3, 5]. Histological studies clearly show that rough surfaces, when compared to smooth ones, can stimulate a faster and effective osseointegration [6–8]. These features were ratified by several clinical studies, proving excellent long-term survival/success rates for implants with modified rough surfaces [9, 10].

Traditional manufacturing and postprocessing methods, however, provide us with fixtures characterized by a high-density titanium core with different micro- or nanorough surfaces [2–4]. Using these methods, it is not possible to fabricate implants with structure possessing a gradient of porosity perpendicular to the long axis and therefore with a highly porous surface and a highly dense core [11, 12].

However, structures with controlled variable porosity can balance the mismatch between different elastic modulus of bone tissues and titanium implants, thus reducing stresses under functional loading and promoting long-term fixation stability and clinical success [11, 12]. Conventionally, cpTi implants present a higher rigidity than surrounding bone because of Young's modulus (elastic modulus) of the material and the geometry of the structure [12]. Elastic modulus of cpTi (112 GPa) and titanium alloy Ti-6Al-4V (115 GPa) are both higher than those of cortical bone (10–26 GPa) [12]. In addition, osseointegration of the dental implant can be biologically improved by a porous structure with an open interconnected pore system; this system can promote bone ingrowth into the metal framework, giving a strong mechanical interlocking between the fixture and the bone [12, 13].

Because of these considerations, there is a demand for new fabrication methods, with the aim of obtaining porous titanium framework, with controlled porosity, pore size, and localization [13, 14].

Porous titanium implants have been introduced in orthopaedics and dental practice since the end of the 1960s with interesting results [15, 16]; however, these were generally obtained using sprays techniques and coating on implant surfaces [16]. However, fatigue resistance of coated implants fabricated with this method may be reduced up to 1/3 when compared with standard uncoated implants [17]. More recently, different fabrication methods to obtain porous titanium frameworks have been proposed including cosintering precursor particles, powder plasma spraying over a high-density core, titanium fibers sintering, and solid-state foaming by expansion of argon-filled pores [17–19]. However, none of these methods can realize titanium scaffolds allowing complete control on the external shape geometry as well as interconnected pore system [12].

In the last few decades, 3D printing/additive manufacturing (3DP/AM) technologies have become more and more important in the world of industry: these allow realizing physical objects starting from virtual 3D data project, without intermediate production steps, saving time and money [12, 20, 21]. With 3DP/AM, porous titanium implants for medical applications can be fabricated. In fact, a high power focused laser beam fuses metal particles arranged in a powder bed and generates the implant layer-by-layer, with no postprocessing steps required [20, 21].

The physical and chemical properties of 3DP/AM titanium have been extensively studied [11, 12, 21]. At a later stage, different* in vitro* studies have investigated the cell response to the surface of 3DP/AM implants, examining the formation of human fibrin clot [22] and the behaviour of human mesenchymal stem cells and human osteoblasts [22, 23]. Several animal [24, 25] and human [26–28] histologic/histomorphometric studies have documented the bone response after the placement of 3DP/AM titanium implants. However, only a few clinical studies have investigated the performance of 3DP/AM titanium dental implants: these are based on a limited number of patients with a short follow-up [29–32].

Hence, the aim of the present prospective clinical study with 3 years of follow-up was to evaluate the survival and success rates of single 3DP/AM titanium dental implants placed in both jaws.

## 2. Materials and Methods

### 2.1. Inclusion and Exclusion Criteria

The present investigation was designed as a prospective multicenter clinical study. Between January 2010 and January 2012, all patients with a single-tooth gap or in need of replacement of a failing, nonrecoverable single tooth, who were referred to 4 different private practices for treatment with dental implants, were considered for enrollment in the present study. Inclusion criteria were good oral health and sufficient bone availability to receive a fixture of at least 3.3 mm in diameter and 8.0 mm in length. Exclusion criteria were poor oral hygiene, nontreated periodontal disease, smoking, and bruxism. The study protocol was exposed to each subject before enrollment: everybody accepted it and signed an informed consent form. The work was performed in accordance with the principles outlined in the Declaration of Helsinki on experimentation involving human subjects, as revised in 2008.

### 2.2. Additive Manufacturing Implants and Characterization

The implants used in this study (Tixos®, Leader Implants, Milan, Italy) were fabricated with an additive manufacturing (AM) technology, starting from powders of titanium alloy (Ti-6Al-4V) with a particle size of 25–45 *μ*m. The implants were fabricated layer-by-layer by an Yb (ytterbium) fiber laser system (EosyntM270®, EOS GmbH, Munich, Germany), operating in an argon controlled atmosphere, using a wavelength of 1,054 nm with a continuous power of 200 W at a scanning rate of 7 m/s and with the capacity to build a volume of 250 × 250 × 215 mm. Laser spot size was 0.1 mm. Postproduction steps consisted of sonication for 5 min in distilled water at 25°C, immersion in NaOH (20 g/L) and hydrogen peroxide (20 g/L) at 80°C for 30 min, and then further sonication for 5 min in distilled water. The implants were then acid-etched in a mixture of 50% oxalic acid and 50% maleic acid, at 80°C for 45 min, and washed for 5 min in a sonic bath of distilled water. These procedures were needed to remove any residual nonadherent titanium particle. The AM implants featured a porous surface with *R*_*a*_ value of 66.8 *μ*m, *R*_*q*_ value of 77.55 *μ*m, and *R*_*z*_ value of 358.3 *μ*m, respectively.

**Figure 1:**
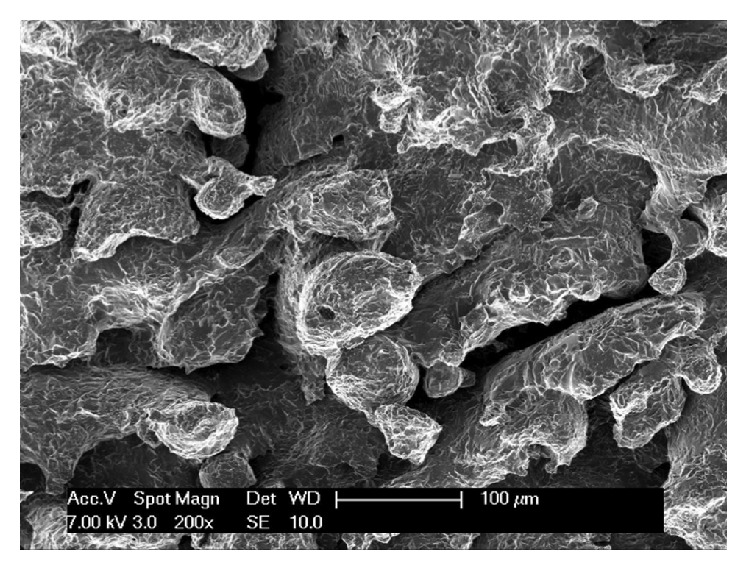
Scanning electron microscopy (SEM) of the 3DP/AM porous implant surface.

The implant surface microstructure consisted of roughly spherical particles ranging between 5 and 50 *μ*m. After exposure to hydrofluoric acid some of these were removed and the microsphere diameter then ranged from 5.1 *μ*m to 26.8 *μ*m. Particles were replaced by grooves with 14.6 to 152.5 *μ*m in width and 21.4 to 102.4 *μ*m in depth after an organic acid treatment. The metal core consisted of prior beta grains. The titanium alloy was composed of titanium (90.08%), aluminium (5.67%), and vanadium (4.25%) (Figure 1). Young's modulus of the inner core material was 104 ± 7.7 GPa, while that of the outer porous material was 77 ± 3.5 GPa. The fracture face showed a dimpled appearance typical of ductile fracture [12].

### 2.3. Preoperative Evaluation

An accurate preoperative evaluation of the oral hard and soft tissue was performed in each patient. Preoperative procedures included the clinical and radiographic examination of the single-tooth gaps. Panoramic and periapical radiographs were taken as primary investigation. In some cases, cone-beam computed tomography (CBCT) was required. CBCT data were processed by dedicated DICOM (Digital Imaging and Communications in Medicine) viewer softwares in order to realize a 3D reconstruction of maxillary bones. With these types of software, it is possible to navigate between maxillary structures and to correctly assess bone features for each implant site, such as thickness, density of the cortical plates and of the cancellous bone, and ridge angulations. Finally, impressions were taken and stone casts were made for the diagnostic wax-up.

### 2.4. Implant Placement

Local anaesthesia was obtained, infiltrating 4% articaine with 1 : 100.000 adrenaline. In patients with a missing single tooth, a crestal incision and two releasing incisions, on mesial and distal sides, were made at surgical site. Full-thickness flaps were elevated depicting alveolar ridge. The preparation of fixture sites was realized with spiral drills of increasing diameter, under constant irrigation with sterile saline. Cover screws were screwed on the implants; then flaps were repositioned using interrupted sutures. In patients with a failing, nonrecoverable single tooth, a flapless approach was followed. A gentle extraction was performed, avoiding any damage of the socket bone walls, using a periotome. Then, the preparation of the surgical site was based on the receiving site's bone quality. The preparation was deepened 3-4 mm apically to the end of the postextraction socket in order to better engage the implant. The implant was placed in position with its cover screw; then particulate bone grafts were placed to fill the space between the implant and the socket walls. Finally, platelet rich in growth factors (PRGF) was prepared, positioned, and sutured to cover the socket in order to protect the surgical site and to accelerate soft tissue healing.

### 2.5. Postoperative Treatment

Pharmacological postsurgery procedures consisted of oral antibiotics 2 g each day for 6 days (Augmentin®, GlaxoSmithKline Beecham, Brentford, UK). Any postoperative pain was managed by administering 100 mg nimesulide (Aulin®, Roche Pharmaceutical, Basel, Switzerland) every 12 h for 2 days. In addition, patients were educated about oral hygiene maintenance with mouth rinses with 0.12% chlorhexidine (Chlorexidine®, OralB, Boston, MA, USA) administered for 7 days. Sutures were removed at 8–10 days after surgery.

### 2.6. Healing Period

Implants were placed with a two-stage technique, waiting a healing period of at least 2-3 months in the mandible and 3-4 months in the maxilla. During the second-stage surgery, underlying fixtures were exposed with a small crestal incision and healing transmucosal abutments were screwed replacing the cover screws. Flaps were stabilized around healing abutment by suturing. Two weeks later, the final impressions were taken, and therefore the final abutments and the provisional crowns were delivered to patients. Provisional crowns, made in acrylic resin, had the task of evaluating the stability of implants under a progressive functional load and influencing the maturation of soft tissues around fixtures before the implementation of final restorations. The provisional crowns were left* in situ* for three months; then the final metal-ceramic crowns were delivered and cemented with zinc phosphate cement or zinc-eugenol oxide cement.

### 2.7. Clinical, Prosthetic, and Radiographic Evaluation

All patients were enrolled in a follow-up recall program, with sessions of professional oral hygiene every 6 months. During these sessions, every year, the implant-supported restorations were carefully checked. Static and dynamic occlusion was controlled, and periapical radiographs were taken using a Rinn alignment system (Rinn®, Dentsply, Elgin, IL, USA). Customized positioners were also used for correct repositioning and stabilization of radiographic template.

At the end of the study, after three years of functional loading, the following clinical, prosthetic, and radiographic parameters were evaluated for each implant.

*Clinical Parameters*

- Presence/absence of pain, sensitivity.
- Presence/absence of suppuration, exudation.
- Presence/absence of implant mobility.

*Prosthetic Parameters*

- Presence/absence of mechanical complications (i.e., complications of prefabricated implant components, such as abutment screw loosening, abutment screw fracture, abutment fracture, and implant fracture).
- Presence/absence of technical complications (i.e., complications related to superstructures, such as loss of retention and ceramic/veneer fracture).

*Radiographic Parameters*

- Presence/absence of continuous peri-implant radiolucency.
- Distance between the implant shoulder and the first visible bone-implant contact (DIB).

This last value, measured with the aim of an ocular grid (4.5x), represented the quantification of crestal bone reabsorption after 3 years of functional loading, and it was measured on mesial and distal side of implant. To compensate for radiographic distortion, the actual (known) fixture length was compared to the radiographic length, using a proportion.

### 2.8. Implant Survival and Implant-Crown Success Criteria

An implant was categorized as survival if it was still in function after three years of functional loading. On the contrary, implant losses were all categorized as failures. Implant mobility in the absence of clinical signs of infection, persistent and/or recurrent infections (with pain, suppuration, and bone loss), progressive marginal bone loss caused by mechanical overload, and implant body fracture were conditions for which implant removal could be indicated. Implant failures were divided into “early” (before the abutment connection) or “late” (after the abutment connection) failures.

An implant-supported restoration was considered successful when the following clinical, prosthetic, and radiographic success criteria were fulfilled:

- Absence of pain, sensitivity.
- Absence of suppuration, exudation.
- Absence of clinically detectable implant mobility.
- Absence of continuous peri-implant radiolucency.
- DIB < 1.5 mm after the first year of functional loading (and not exceeding 0.2 mm in each subsequent year).
- Absence of prosthetic (mechanical or technical) complications.

## 3. Results

Eighty-two patients (44 males, 38 females; age range 26–67 years), who were recruited in 4 different clinical centers, fulfilled the inclusion criteria and did not present any of the conditions enlisted in the exclusion criteria: therefore, they were enrolled in the present study. In total, 110 implants (65 maxilla, 45 mandible) were installed: 75 in healed ridges and 35 in postextraction sockets. The prosthetic restorations included 110 single crowns (SCs): 32 of these were in the anterior areas (incisors, cuspids, and first premolars), while 78 were in the posterior areas (second premolars, molars). Lengths and diameters of used implants were summarized in Table 1.

**Figure 2:**
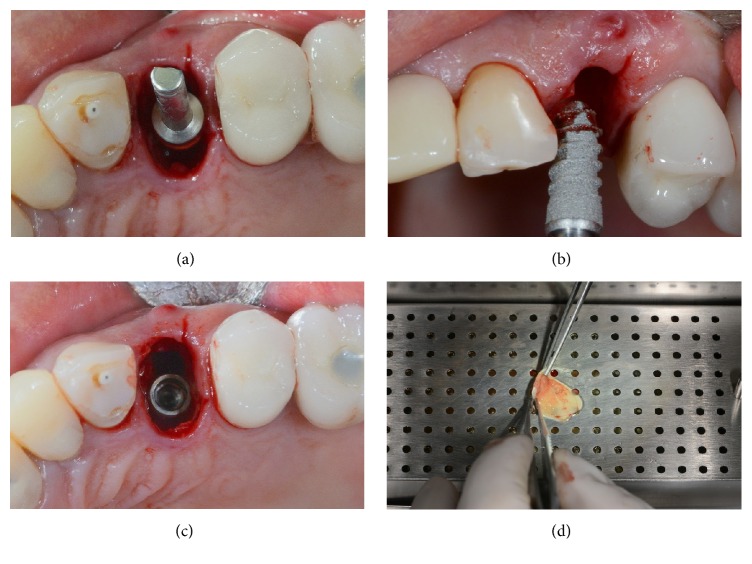
Placement of a single 3DP/AM titanium dental implant in a postextraction socket of the posterior maxilla: surgical phases. (a) Preparation of the implant site. (b) Placement of the 3DP/AM porous implant in the postextraction socket. (c) The implant in position. (d) Preparation of the biological membrane-platelet rich in growth factors (PRGF).

**Figure 3 fig3:**
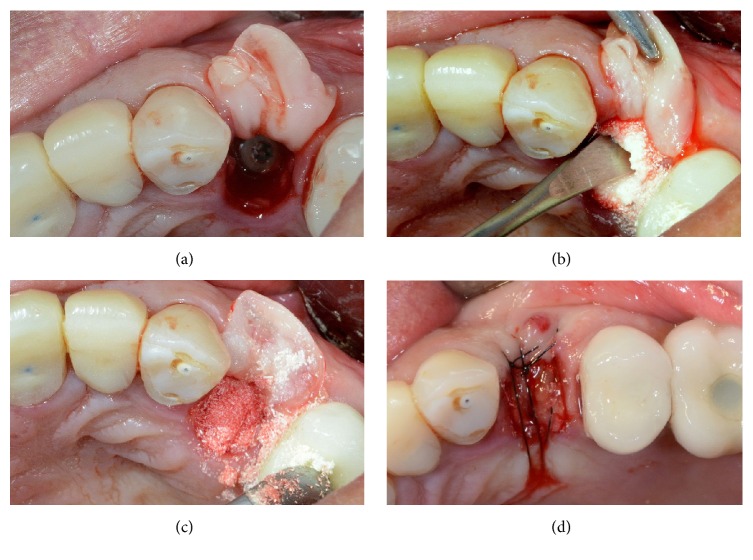
Placement of a single 3DP/AM titanium dental implant in a postextraction socket of the posterior maxilla: surgical phases. (a) The biological membrane is ready to be sutured for protecting the socket. (b) Socket preservation with particulate bone grafts. (c) The socket is completely filled with particulate bone grafts before the sutures. (d) Sutures.

**Figure 4 fig4:**
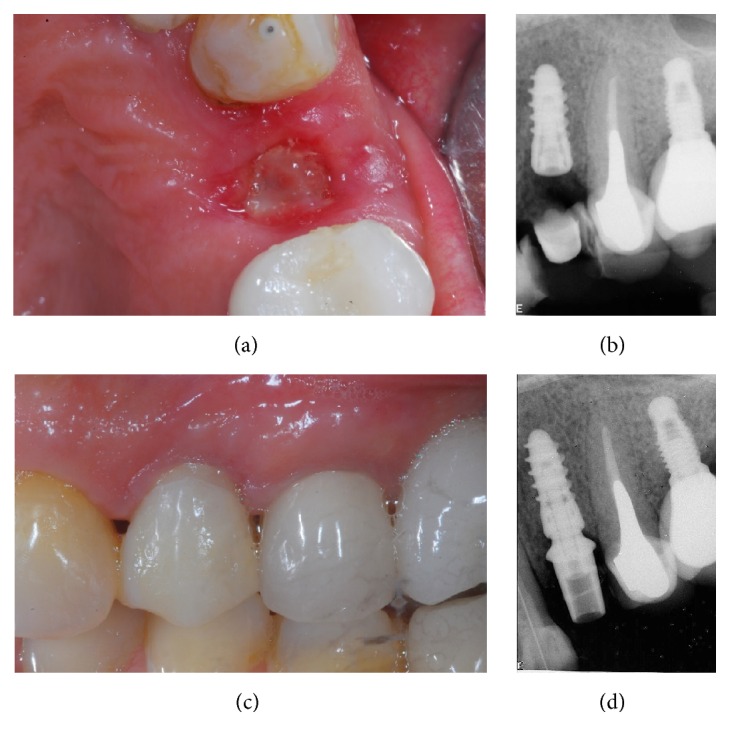
A single 3DP/AM titanium dental implant in a postextraction socket of the posterior maxilla: healing phases. (a) Ten days after surgery, sutures are removed. (b) Periapical rx 10 days after implant placement. (c) Three months later, a provisional restoration is placed. (d) Periapical rx at placement of the provisional restoration.

**Figure 5:**
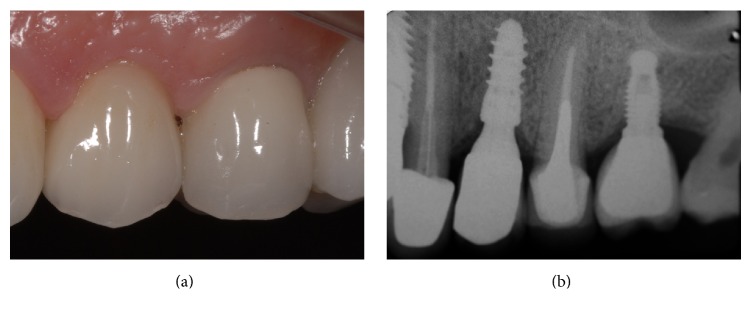
A single 3DP/AM titanium dental implant in a postextraction socket of the posterior maxilla: 3-year follow-up control. (a) Clinical picture after 3 years of functional loading. (b) Periapical rx after 3 years of functional loading.

At the end of the study, after 3 years of functional loading, six implants failed (four during the healing period and before the abutment connection, because of implant mobility in the absence of clinical signs of infection, two after the abutment connection, one for persistent/recurrent peri-implant infection, and another one for implant body fracture) for an overall implant survival rate of 94.5% (Figures 2 [Fig fig3] [Fig fig4]–5). Four implants failed in healed ridges, whereas two implants failed in extraction sockets. In the maxilla, four implants failed (3 implants showed mobility and lack of osseointegration in absence of infection, in the posterior maxilla, and had to be removed; an implant body fracture occurred in the anterior maxilla) for a survival rate of 93.8%; in the mandible, two implants failed (one for lack of osseointegration and another one for persistent/recurrent infection, both in the posterior mandible) and were removed, for a survival rate of 95.6%.

With regard to the implant-crown success, no implants showed pain or sensitivity, suppuration or exudation, or continuous peri-implant radiolucency. However, two implants showed a DIB > 1.5 mm during the first year of functional loading and were therefore considered not successful; in addition, three prosthetic abutments became loose, in the posterior areas of the mandible. These abutments were reinserted and screwed again; however, these were considered prosthetic complications. Another prosthetic complication registered was a ceramic chipping in a maxillary molar. At the end of the study, among the 104 surviving implant-supported restorations, 6 showed complications and were therefore considered unsuccessful, for a 3-year implant-crown success of 94.3%. Finally, the mean DIB was 0.75 mm (±0.32) and 0.89 (±0.45) after 1 year and 3 years of functional loading, respectively.

## 4. Discussion

A porous structure has many biological advantages. In fact, it facilitates the diffusion of biological fluids and nutrients for the maturation of the tissues and the removal of waste products of metabolism; moreover, it allows cell ingrowth and reorganization as well as neovascularization from surrounding tissues. A scaffold with well-defined porosity characteristics (pores size, geometry, distribution, and interconnectivity) can therefore enhance bone ingrowth [12, 13, 20, 33]. In this context the size of the interconnections between pores, according to several researchers, seems to be one of the most important parameters influencing the bone growth in its structure [20].

According to some researchers, pore size between 200 and 400 *μ*m seems to be the ideal measure to positively influence the behaviour of bone cells [20, 33], whereas Sachlos and Czernuszka [34] have achieved excellent results using a scaffold with 500 *μ*m pores size. Xue et al. [35, 36] have recently assessed the* in vitro* reaction of bone cells in presence of a porous titanium AM scaffold. The authors have highlighted how osteoblasts spread on the surface, migrate into the cavities of these porous scaffolds (pore sizes of 200 *μ*m or higher are recommended), and produce new bone matrix [35, 36]. The* in vivo* physiological response to these porous scaffolds includes the formation of new tissue that infiltrates the network, with capillaries, perivascular tissues, and progenitor cells migrating into the pore system and supporting the healing processes [35, 36]. Because of the considerable amount of data in the current literature, there is still no agreement on the optimal size of the pores for endosseous implants; however, pore sizes between 100 and 400 *μ*m seem to be able to support the formation of mineralized bone inside porous scaffolds [37, 38].

Although the benefits of an open-pore structure with controlled porosity at the implant surface have been elucidated [38], it was very difficult to realize implants with these characteristics using standard production methods.

3DP/AM techniques have been recently proposed in order to overcome these obstacles and to fabricate endosseous implants (including dental implants) with controlled and functionally graded porosity [11–13]. 3DP/AM is able to control the porosity of each layer and consequently the porous structure of the whole implant by simply modifying some processing parameters (such as power and diameter of the focused laser beam, layer thickness and distance between them, the size of the original titanium powders, and processing atmosphere) [11, 12]. With this method, it is also possible to control the size, distribution, and interconnectivity of pores [13], giving a controlled, open-pore network. In addition, 3DP/AM allows implants with a gradient of porosity along the main axis to be fabricated [12]. Finally, 3DP/AM implants do not require postfabrication process: they do not require decontamination, since they are not machined and therefore no oils or contaminants are employed. Moreover, they do not need surface treatments, and this may further reduce the costs.

One of early steps of osseointegration process involves migration of osteoprogenitor cells into fibrin network established on the implant surface [12, 13, 20]. A recent* in vitro* study [22] reported that human fibrin can quickly realize, around porous AM titanium surface, a stable three-dimensional network. Moreover, AM porous titanium surfaces are able to recruit osteoprogenitor cells that, when differentiated into osteoblasts, produce woven bone under the influence of bone morphogenetic proteins, vascular endothelial growth factor, and other specific bone proteins [22, 23]. Research has partially clarified some of the mechanisms that regulate cell functions and differentiation. Cells interact with their substrates through specific adhesion membrane proteins, called integrins, which are responsible for the formation of focal adhesion plaque [38–40]. Moreover, integrins are linked to specific cytoskeleton adaptor proteins through their cytoplasmic domain. The formation of focal adhesion plaques and subsequent cell adhesion generate mechanical forces that are converted into biochemical signals within cells by integrins and other mechanoreceptors [38–40]. Thus, the geometry of substrates can affect a wide spectrum of cellular responses [38–40]. Geometric properties of implant surface are then able to induce modification in cell shape, inducing changes on genes expression [39].

The surface generated with 3DP/AM technology, characterized by pores, cavities, and interconnections, could represent a powerful stimulus for osteogenic phenotype expression, as demonstrated in different* in vitro* studies [21, 22]. Cells in contact with AM surfaces are forced to take specific three-dimensional shape according to scaffold pores and cavities, generating mechanical stresses that induce osteogenic phenotype expression [22, 23].

All these findings have been confirmed in a series of histologic and histomorphometric studies, in animals [24, 25] and humans [26–28]. However, until now, only a few clinical studies have dealt with 3DP/AM titanium implants [29–32].

In a first multicenter clinical study evaluating the survival and success of 201  3DP/AM porous titanium implants supporting fixed restorations (single crowns, fixed partial prostheses, and fixed full arches), 201 implants were inserted in 62 subjects [29]. Most of the implants (122) were placed in the posterior areas of jaws. After 1 year of loading, an overall implant survival rate of 99.5% was reported, with only one failed and removed fixture [29]. In this first study, among the surviving fixtures implants (200), only 5 could not satisfy the success criteria, for an implant-crown success of 97.5% [29]; moreover, a mean distance between the implant shoulder and the first bone-to-implant contact of 0.4 mm (±0.2) was reported [29].

Another clinical study aimed at evaluating survival, complications, and peri-implant marginal bone loss of 3DP/AM porous titanium implants used to support bar-retained maxillary overdentures [30]. Over a 2-year period, 120 fixtures were installed in the maxilla of 30 subjects to support bar-retained overdentures. Each denture was supported by 4 splinted implants, by means of a rigid cobalt chrome bar. The patient-based implant survival and incidence of biologic and prosthetic complications were registered. At the 3-year follow-up examination, three implants failed and had to be removed, for an overall survival rate of 92.9% [30]. The biologic complications amounted to 7.1%, whereas the prosthetic complications were more frequent (17.8%) [30]. At the 3-year examination, the peri-implant marginal bone loss amounted to 0.62 mm (±0.28); therefore the authors concluded that the use of 4  3DP/AM titanium implants to support bar-retained maxillary overdentures can be considered a safe and successful treatment procedure [30].

Finally, in a recent previous clinical study, 231 one-piece 3DP/AM porous titanium mini-implants (2.7 and 3.2 mm diameter) were inserted in 62 patients to support immediately loaded mandibular overdentures [31]. In this study, six fixtures failed after a period of 4 years of functional loading, giving an overall cumulative survival rate of 96.9% [31]. The biologic complications amounted to 6.0%, while the prosthetic complications were more frequent (12.9%). Finally, a mean DIB of 0.38 mm (±0.25) and 0.62 mm (±0.20) was reported at the 1-year and 4-year follow-up examinations, respectively [31].

These results seem to be in accordance with those of our present 3-year follow-up prospective clinical study, in which the clinical behaviour of 110 single implants produced with 3DP/AM technology and placed in both jaws was evaluated. A satisfactory survival rate was observed (94.5%), with only six failed and removed implants. Among the 104 implants still in function at the end of the follow-up period, 98 were categorized as successful, giving an implant-crown success rate of 94.3%. Only six implants could not attain implant-crown success criteria: two fixtures displayed a DIB > 1.5 mm after 1 year of functional loading, three implants presented loosening of prosthetic abutment during follow-up period, and another implant-supported restoration had a ceramic chipping. Furthermore, radiographic evaluation showed an excellent bone stability around single 3DP/AM implants. The mean distances between the implant shoulder and the first visible bone-to-implant contact (DIB) were 0.75 mm (±0.32) and 0.89 (±0.45) after 1 year and 3 years of functional loading, respectively.

## 5. Conclusions

In this 3-year follow-up prospective clinical study, single 3DP/AM implants have shown 94.5% of survival rate and 94.3% of implant-crown success rate. Considering these results, dental implants produced with 3DP/AM technologies seem to represent a successful clinical option for the rehabilitation of single-tooth gaps in both jaws, at least after a 3-year follow-up. However, the present study has clear limits (such as limited number of patients treated and fixtures installed and short follow-up period); therefore further long-term clinical studies will be necessary to evaluate the long-term performance as well as the mechanical resistance of single 3DP/AM implants placed in both jaws. In addition, real potential of 3DP/AM implants in restoring partially or completely edentulous arches still needs to be elucidated.

## Figures and Tables

**Table 1:** Implant distribution by length and diameter.

	8.0 mm	10.0 mm	11.5 mm	13.0 mm	
3.3 mm	—	5	5	5	15
3.75 mm	2	40	23	3	68
4.5 mm	4	10	13	—	27
	6	55	41	8	110
